# Long-term follow-up of cervical disease in women screened by cytology and HPV testing: results from the HART study

**DOI:** 10.1038/sj.bjc.6605619

**Published:** 2010-03-30

**Authors:** D Mesher, A Szarewski, L Cadman, H Cubie, H Kitchener, D Luesley, U Menon, G Hulman, M Desai, L Ho, G Terry, A Williams, P Sasieni, J Cuzick

**Affiliations:** 1 Cancer Research UK Centre for Epidemiology, Mathematics & Statistics, Wolfson Institute of Preventive Medicine, Queen Mary University of London, Barts & The London School of Medicine and Dentistry, Charterhouse Square, LondonEC1 M 6BQ, UK; 2 Royal Infirmary of Edinburgh, 51 Little France Crescent, Edinburgh, EH16 4SA, UK; 3 University of Manchester, Oxford Road, Manchester, M13 9PL, UK; 4 Pan-Birmingham Gynaecological Cancer Centre, City Hospital, Dudley Road, Birmingham, B18 7QH, UK; 5 Gynaecological Cancer Research Centre, Institute for Women's Health, University College London, 1st Floor maple House, 149 Tottenham Court Road, London W1T 7DN, UK; 6 Department of Histopathology, The King's Mill Centre, Mansfield Road, Sutton-in-Ashfield, Nottinghamshire NG17 4JL, UK; 7 Manchester Cytology Centre, Clinical Sciences Building 2, Oxford Road, Manchester Royal Infirmary, Manchester, M13 9WW, UK

**Keywords:** HPV testing, cytology, cervical screening, long-term follow-up

## Abstract

**Background:**

Several studies have shown that testing for high-risk human papillomavirus (HPV) types results in an improved sensitivity for CIN2+, compared with cytology, although with a somewhat lower specificity.

**Methods:**

We obtained follow-up results, with at least one smear after participation in the HART study, which compared HPV testing (HC-II) with cytology as a primary screening modality.

**Results:**

With a median follow-up of 6 years, 42 additional cases of CIN2+ were identified; women who were HPV positive at baseline were more likely to develop CIN2+ than those who were HPV negative (hazard ratio (HR) 17.2; 95% confidence interval (CI) (9.3–31.6)) and the risk increased with increasing viral load. Compared with HPV-negative women (relative light unit (RLU) <1), the HR (95% CI) was 5.4 (1.6, 18.2) for 1–10 RLU and 25.5 (13.6, 47.9) for RLU ⩾10. Positive cytology (borderline or worse compared with negative) was also predictive of developing CIN2, although to a lesser extent (HR 8.7; 95% CI (4.5–17.1)). Only one case of CIN3 and three cases of CIN2 were found in women who showed a positive cytology result but were HPV negative at baseline.

**Conclusion:**

After 5 years of follow-up, CIN2+ occurred in 0.23% of women who were HPV negative at baseline compared with 0.48% of women who showed a negative cytology result, indicating a much longer low-risk interval for CIN2+ after HPV testing.

The introduction of cervical screening programmes based on cytology has led to a substantial reduction in the incidence of cervical cancer in countries in which it has been adequately organised (Laara *et al*, 1987; Sasieni *et al*, 2003; Bray *et al*, 2005; van der Aa *et al*, 2008). However, cytology is a subjective test with comparatively low sensitivity (50–75%) to detect CIN2 or worse (CIN2+) (Cuzick *et al*, 2006). Hence, regular screens at relatively short intervals are required. High-risk types of the human papillomavirus (HPV) are present in almost all cervical cancers (Clifford *et al*, 2006), and it has been shown in several studies that testing for these high-risk HPV types results in an improved sensitivity for CIN2+ compared with cytology, although at a somewhat lower specificity (especially in younger women) (Cuzick *et al*, 2006; Leinonen *et al*, 2009). Recently published results have reported that a negative HPV result is associated with a greater long-term protection from CIN2+ compared with a normal cytology result (Bulkmans *et al*, 2007; Cuzick *et al*, 2008; Dillner *et al*, 2008).

In this study, we report results on the long-term follow-up of the HART study (Cuzick *et al*, 2003) and examine long-term protection after a negative HPV test result alone compared with a negative cytology result, or a negative result on both tests.

## MATERIALS AND METHODS

A total of 11 085 women, aged 30–60 years, were recruited from 161 family practices associated with five UK laboratories or referral centres in Birmingham, Edinburgh, London, Manchester and Mansfield. Women were recruited and attended an initial visit between June 1998 and July 2001. Women were excluded if they had had an abnormal smear within the last 3 years or had been previously treated for CIN. At the initial baseline visit, two cervical samples were taken; the first, for conventional cytology, was taken with an extended-tip spatula, the second sample was taken using a cone-shaped cervical sampler brush (Qiagen, Hilden, Germany), which was placed into specimen transport medium for transportation to one of two laboratories. This second sample was tested for high-risk HPV types (16, 18, 31, 33, 35, 39, 45, 51, 52, 56, 58, 59 and 68) using the Digene (now Qiagen) Hybrid Capture II kit (HC-II). Patients were considered HPV positive if their result was above the 1 relative light unit (RLU) control value, in keeping with the manufacturer's instructions. Risk associated with increasing RLU levels was also investigated to determine the impact of viral load.

Women with mild dyskaryosis or worse or with two to three inadequate cytology results were referred to colposcopy. Women with a borderline smear or an HPV-positive result (or both) were randomised into two equal groups to receive immediate colposcopy or a further cytology and HPV test in 6–12 months. Those with both a negative cytology and a negative HPV test result were considered disease free and continued routine screening. However, 460 of these women were randomly selected for colposcopy to ascertain disease rates in this subgroup of women.

Full details of testing and management of women at baseline are given in the baseline paper (Cuzick *et al*, 2003).

All women were followed up to determine results of subsequent routine cytology screening. Any ensuing colposcopy and biopsy results were also obtained. Follow-up commenced at the date of the baseline HART test and continued until the last recorded smear, which varied between December 2006 and March 2008 according to centre. Any discrepancies between baseline and follow-up data were checked at the relevant cytology/histology laboratories where possible. If we were unable to verify the result at the laboratory, then the most recent results from the centre were used.

Follow-up cytology was obtained using either an internally developed computer system (Mansfield, Manchester, Edinburgh), the national computerised call/recall NHAIS system (usually referred to as Open Exeter) (London) or a combination of both systems (Birmingham, with discrepancies being resolved by a review of laboratory records). Follow-up colposcopy and histology results were obtained for each centre using their internal computer systems.

Criteria for referral to colposcopy varied between different sites: Birmingham – one mild dyskaryosis or worse; three borderline or inadequate smears. After biopsy, all women with high-grade disease were treated, and some cases of low-grade disease as well. Edinburgh – one moderate dyskaryosis or worse, or glandular abnormality; two mild dyskaryosis; three borderline; three inadequate smears. An option of treatment or conservative management was offered to patients with biopsy-confirmed CIN1 and all CIN2 or worse cases were offered treatment. London – one mild dyskaryosis or worse; three borderline or inadequate smears. An option of treatment or conservative management was offered to patients with biopsy-confirmed CIN1 and all CIN2 cases or worse were offered treatment. Manchester – one mild dyskaryosis or worse; three borderline or inadequate smears. After biopsy, all cases of high-grade and some cases of low-grade disease were treated. Mansfield – two mild dyskaryosis or worse; two borderline or three inadequate smears. After biopsy, all cases of high-grade and some cases of low-grade disease were treated.

Additional ethical approval for the follow-up stage of the study was obtained by the MREC involved with the original HART study (Cuzick *et al*, 2003). Approval was given for passive outcome follow-up of women attending routine screening, but a pathology review of biopsies was not performed because of concerns about the need to contact women if discrepancies were found.

### Statistical methods

All eligible women from the baseline study were included (Cuzick *et al*, 2003). Baseline test results were updated on the basis of additional data available for the first year, which became available after publication. These updated results were used to recalculate sensitivity and specificity for CIN2 or worse (and CIN3 or worse) within the first year for cytology and HPV. For follow-up results, we included only women with at least one follow-up cytology result at least 1 year after the initial baseline test. The main outcome of interest was the presence of CIN2+ on histology after the initial year. We also considered CIN3+ as a further outcome. Histological events were backdated to the time of the preceding cytology, and follow-up time at risk was taken from the date of the initial baseline test until the date of the last cytology test recorded for that individual. Outcomes were assessed by hazard ratios (HRs) for time to event using the proportional hazards model. All *P*-values are two sided. Statistical analyses were performed using STATA (StataCorp. 2007. Stata Statistical Software: Release 10; StataCorp LP, College Station, TX, USA).

## RESULTS

Initial results for the baseline period have been published (Cuzick *et al*, 2008), and have been updated to reflect additional histology results within a year of the baseline visit (and additional cytology within 1 year in cases in which only inadequate results were previously available). Seventeen additional women with CIN2+ were identified: one woman (CIN2) had no adequate cytology within the first year but had an HPV-negative result; four women (CIN3) had negative results for both cytology and HPV and the remaining 12 women (six CIN2, six CIN3) had HPV-positive results and a borderline or worse cytology. We found 107 women with histologically confirmed CIN2 or worse within a year of this visit. Table 1 shows the level of high-grade disease within a year of the baseline test according to baseline HPV and cytology results. Results were similar to those previously published, with a sensitivity of HPV for CIN2+ of 92.5% (*vs* 97.1% previously) and for cytology of 82.1% (*vs* 76.6% previously). For CIN3+, sensitivity was 96.1% for HPV (97.1% previously) and 82.9% for cytology (82.6% previously).

**Figure 1:**
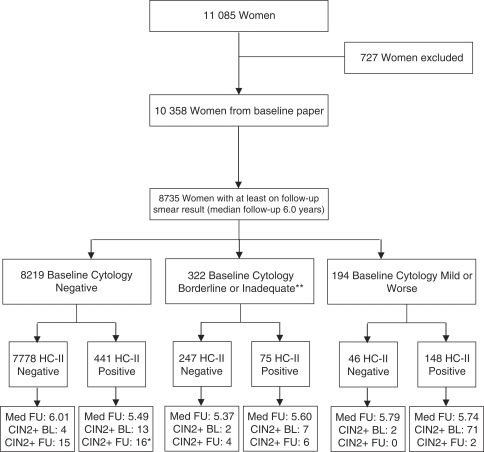
The HART trial profile. ^*^Includes 1 woman who had hgd at baseline and during follow-up. ^**^53 women had no adequate Cytology within one year of baseline.

The trial profile (Figure 1) shows the number of women followed up and the proportions with high-grade disease after the first year. Follow-up time was defined as time between baseline test and last subsequent smear test (any colposcopy or histology after the last smear test has been included but the last follow-up date is still considered as the date of cytology). A total of 8735 (84%) women had at least one further smear recorded 1 year or more after entry, with a median follow-up of 6 years (Birmingham: 1535 women, 6 years; Edinburgh: 1620 women, 5.6 years; London: 2194 women, 6.3 years; Manchester: 1075 women, 4.6 years; Mansfield: 2311 women, 6 years).

During follow-up, a further 42 cases of CIN2+ were found. Table 2 shows the worst histology for each woman after the baseline year. Four cases of CIN2+ were found during the follow-up period among those who were HPV negative but cytology positive. Three of these were CIN2, and they occurred in women with borderline cytology between 19 and 52 months after entry. One case of CIN3 occurred at 44 months, also after a borderline entry smear. Sixteen cases of CIN2+ were found on follow-up in women who were HPV positive but cytology negative (12 CIN2, 4 CIN3+) and 15 cases of CIN2+ were found in women who were negative on both tests (seven CIN2, six CIN3+).

Of the 65 women with no adequate cytology in the first year from the baseline test, 53 (81.5%) had a further smear more than 1 year after baseline (50 HPV negative and 3 positive), but among them, no high-grade disease was identified. Three women had an ungraded CIN at some stage in their follow-up, of whom two had an additional histological diagnosis within a period of 2 months, which confirmed CIN3 (both HPV positive, one cytology normal and one cytology moderate at baseline). The remaining woman had no further follow-up recorded and this result was taken to be CIN2 (cytology normal and HPV negative at baseline).

### Incidence of CIN2+ by baseline HPV and cytology results

**Figure 2:**
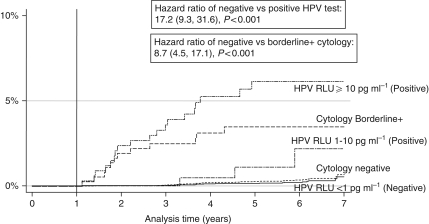
Cumulative incidence of CIN2+ in the follow-up period according to baseline results.

A total of 27 (0.28%) women with a negative HPV test at baseline (*n*=9574) were identified as having CIN2+ at some stage. Eight (30%) of them were identified in the baseline year and the remaining 19 (70%) after this period. Including disease found at baseline, the risk of developing CIN2+ at 1, 3, 5 and 8 years after a negative HPV test result was 0.09, 0.12, 0.23 and 0.61%, respectively. Figure 2 shows the rate of disease restricted to the follow-up period only, being 0.04, 0.15 and 0.53% at 3, 5 and 8 years, respectively, after a negative HPV test result, and 2.14, 4.11 and 6.20%, respectively, after a positive HPV test result (HR 17.16 (95% confidence interval (CI) 9.3, 31.6), *P*<0.0001). There is a clear increase in incidence of CIN2+ as the RLU ratio increases. Compared with an RLU ratio <1, the HR (95% CI) for CIN2+ was 5.4 (1.6, 18.2) for 1–10 RLU and 25.2 (13.6, 47.9) for RLU ⩾10 (test for trend, *P*<0.001).

In all, 49 (0.50%) women with a normal cytology result at baseline (*n*=9782) were identified as having CIN2+ at some stage. Nineteen (39%) of them were identified in the baseline year and the remaining 30 (61%) after this period. Including any disease found at baseline, the risk of developing CIN2+ at 1, 3, 5 and 8 years after a normal cytology result was 0.21, 0.28, 0.48 and 1.04%, respectively. Figure 2 shows the cumulative incidence of new high-grade disease restricted to follow-up only, according to baseline cytology results. In those cytology negative and cytology borderline or worse results, respectively, the levels of CIN2+ were 0.07 *vs* 2.44% at 3 years, 0.28 *vs* 3.40% at 5 years and 0.84 *vs* 3.40% at 8 years (HR 8.74 (95% CI; 4.5, 17.1), *P*<0.0001).

**Figure 3:**
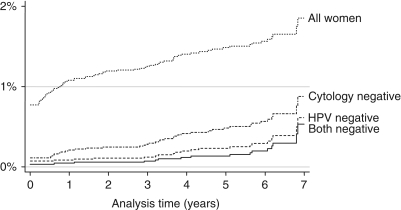
Cumulative incidence of CIN2+ according to baseline results.

Of the 9247 women with both negative cytology and HC-II tests at baseline, 19 (0.21%) had CIN2+, including 4 identified within the first year. Figure 3 shows rates of CIN2+ (including baseline disease) according to baseline cytology and HPV result and shows a condensed scale to allow a comparison of negative cases. Here, we focus on individuals with discordant HPV/cytology results at baseline. For women who were HPV negative but had a borderline or worse cytology result, 4 of 240 (1.67%) were found to have high-grade disease in the follow-up period (rates at 3, 5 and 8 years were 0.43, 1.97 and 1.97%, respectively). For those with a negative cytology but positive HPV test result, 15 of 428 (3.50%) CIN2+ cases were identified and rates at 3, 5 and 8 years were 0.95, 3.60 and 6.20%, respectively.

## DISCUSSION

These results confirm previous reports of a longer low-risk period after a negative HPV test result than after a negative cytology result(Cuzick *et al*, 2008; Dillner *et al*, 2008). It has long been suspected that the higher initial detection with HPV testing should lead to a longer low-risk period in those who are HPV negative. In this multicentre study, we found that women with a negative HC-II test result had a substantially lower rate of CIN2+ for at least 6 years when compared with those with a negative cytology result. This adds more support for the proposition that the interval for screening using HPV could be safely extended to at least 5 years. Further support is provided by a recent Italian study in which invasive cancer was also dramatically reduced in the follow-up period after screening (nine after cytology testing *vs* none after HPV testing) (Ronco *et al*, 2010). A strength of this study is that screening and follow-up processes of these 8735 women were performed in many centres across the United Kingdom as part of the routine screening programme. Cytology and HPV testing was performed on all women in the study, so that comparisons between the two tests could be conducted in a manner that avoided between-women variation. This is ideal for cross-sectional and some longitudinal comparisons, but does not exactly reproduce the results of a randomised trial in which women receive only HPV test or cytology initially and thus would not have disease identified by the other test detected so quickly. Such trials need to be large to be informative, and given the number of smaller trials of HPV testing already reported, it should be large enough to demonstrate a reduction in a more distal end point such as cancer incidence (Sasieni and Cuzick, 2002; Ronco *et al*, 2010).

Women with HPV positive or cytology borderline results (or both) were randomised to either immediate colposcopy or referral to a further smear/HPV test in 12 months. The rate of CIN2+ in the follow-up period was similar regardless of the randomisation group. Therefore, results were unchanged regardless of whether women are referred for immediate colposcopy or managed by follow-up screening. Some overtreatment of lesions with CIN1 (or less) based on the initial biopsy results will lead to lower disease rates during follow-up. We recorded treatment of 17 of 65 (26.2%) women with histologically confirmed CIN1 and 26 of 146 (17.8%) women with a biopsy indicating less than CIN1.

In addition, the histology results in the follow-up phase were not centrally reviewed because of concerns regarding contacting women if there were discrepancies. Therefore, especially for CIN2, which is known to have substantial inter-reader variability and also uncertain progressive potential, it is possible that not all of these were progressive lesions. An exit screen in which all women receive both tests would help to minimise any ascertainment bias. In addition, this study was performed in women aged 30–60 years so that it does not provide any evidence for the relative benefits of cytology *vs* HPV testing in younger women, wherein both tests have a higher positivity rate.

In summary, HPV testing offers improved protection from CIN2+ after a negative test result compared with the protection afforded from a normal cytology result. The risk of developing CIN2+ after a negative HPV test result was extremely low at 5 years (0.23%) and was comparable with cytology at 3 years (0.28%). These results provide additional support for using HPV as the primary screening test, and indicate that the very high sensitivity of HPV testing can not only lead to a more effective screening programme but the resulting high negative predictive value can safely allow longer screening intervals and result in a more cost-effective programme as well.

## Figures and Tables

**Table 1:** Baseline cytology and HPV *vs* worst histology in first year (reviewed histology)

	**Worst histology in first year**							
	**Inadequate**	**Normal/no. of biopsy**	**HPV alone/ borderline**	**CIN1**	**CIN2**	**CIN3**	**Adeno *in situ***	**Total**
*Cytology*								
Inadequate	2	59	3	0	0	1	0	65 (0.6)
Negative	155	9566	27	15	6	13	0	9782 (94.4)
Borderline	24	236	15	13	4	5	0	297 (2.9)
Mild	9	70	18	8	9	8	0	122 (1.2)
Moderate	1	16	1	2	9	13	0	42 (0.4)
Severe	0	3	2	3	2	30	3	43 (0.4)
Inv.Carc/Gland.Neo	0	3	0	0	0	1	3	7 (0.1)
								
*HC-II (RLU ratio)*								
<0.3 (negative)	110	8278	21	18	5	1	0	8433 (81.4)
0.3–0.99 (negative)	17	1115	6	1	0	2	0	1141 (11)
1.0–1.99 (positive)	11	92	3	2	0	1	0	109 (1.1)
2.0–3.99 (positive)	8	79	3	2	1	3	0	96 (0.9)
4.0–9.99 (positive)	7	74	2	2	2	2	0	89 (0.9)
10+ (positive)	38	315	31	16	22	62	6	490 (4.7)
								
Total	191	9953	66	41	30	71	6	10358 (100)
	(1.8)	(96.1)	(0.6)	(0.4)	(0.3)	(0.7)	(0.06)	

Abbreviations: Inv.Carc/Gland.Neo=Possible invasive Carcinoma Glandular Neoplasia; RLU=relative light unit.

**Table 2:** Baseline cytology and HPV *vs* worst follow-up histology (local histology)

	**Worst follow-up histology after first year**							
	**Inadequate**	**Normal/no. of biopsy**	**HPV alone**	**Borderline**	**CIN1**	**CIN2**	**CIN3**	**Total**
*Cytology*								
Inadequate	16	37	0	0	0	0	0	53 (0.6)
Negative	230	7884	24	6	44	13	18	8219 (94.1)
Borderline	75	166	9	0	9	7	3	269 (3.1)
Mild	66	32	1	1	8	1	1	110 (1.3)
Moderate	33	4	2	0	2	0	0	41 (0.5)
Severe	34	2	0	0	2	0	0	38 (0.4)
Inv.Carc/Gland.Neo	4	1	0	0	0	0	0	5 (0.1)
								
*HC-II (RLU ratio)*								
<0.3 (negative)	202	6842	22	5	29	12	6	7118 (81.5)
0.3–0.99 (negative)	32	910	2	0	8	0	1	953 (10.9)
1.0–1.99 (positive)	33	55	0	0	6	0	2	96 (1.1)
2.0–3.99 (positive)	15	58	2	0	1	0	0	76 (0.9)
4.0–9.99 (positive)	17	49	1	0	2	0	1	70 (0.8)
10+ (positive)	159	212	9	2	19	9	12	422 (4.8)
								
Total	458	8126	36	7	65	21	22	8735 (100)
	(5.2)	(93)	(0.4)	(0.08)	(0.7)	(0.2)	(0.3)	

Abbreviations: Inv.Carc/Gland.Neo=Possible invasive Carcinoma Glandular Neoplasia; RLU=relative light unit.
